# The Brain-Gut Axis: Psychological Functioning and Inflammatory Bowel Diseases

**DOI:** 10.3390/jcm10030377

**Published:** 2021-01-20

**Authors:** Spyros Peppas, Claudia Pansieri, Daniele Piovani, Silvio Danese, Laurent Peyrin-Biroulet, Andreas G. Tsantes, Enrico Brunetta, Argirios E. Tsantes, Stefanos Bonovas

**Affiliations:** 1 Department of Gastroenterology, Athens Naval Hospital, 11521 Athens, Greece; peppas.spyros@gmail.com; 2 Department of Biomedical Sciences, Humanitas University, 20090 Milan, Italy; pansieri.claudia@gmail.com (C.P.); sdanese@hotmail.com (S.D.); enrico.brunetta@humanitas.it (E.B.); 3 Humanitas Clinical and Research Center–IRCCS, 20089 Milan, Italy; 4 Department of Gastroenterology, Inserm U1256 NGERE, Nancy University Hospital, Lorraine University, 54500 Vandoeuvre-les-Nancy, France; peyrinbiroulet@gmail.com; 5 Attiko Hospital, School of Medicine, National and Kapodistrian University of Athens, 12462 Athens, Greece; andreas.tsantes@yahoo.com (A.G.T.); atsantes@yahoo.com (A.E.T.)

**Keywords:** neuropsychology, gastroenterology, inflammatory bowel diseases, mental disorders, anti-depressive agents

## Abstract

The brain-gut axis represents a complex bi-directional system comprising multiple interconnections between the neuroendocrine pathways, the autonomous nervous system and the gastrointestinal tract. Inflammatory bowel disease (IBD), comprising Crohn’s disease and ulcerative colitis, is a chronic, relapsing-remitting inflammatory disorder of the gastrointestinal tract with a multifactorial etiology. Depression and anxiety are prevalent among patients with chronic disorders characterized by a strong immune component, such as diabetes mellitus, cancer, multiple sclerosis, rheumatoid arthritis and IBD. Although psychological problems are an important aspect of morbidity and of impaired quality of life in patients with IBD, depression and anxiety continue to be under-diagnosed. There is lack of evidence regarding the exact mechanisms by which depression, anxiety and cognitive dysfunction may occur in these patients, and whether psychological disorders are the result of disease activity or determinants of the IBD occurrence. In this comprehensive review, we summarize the role of the brain-gut axis in the psychological functioning of patients with IBD, and discuss current preclinical and clinical data on the topic and therapeutic strategies potentially useful for the clinical management of these patients. Personalized pathways of psychological supports are needed to improve the quality of life in patients with IBD.

## 1. Introduction

The brain-gut axis represents a complex bi-directional system comprising multiple interconnections between the neuroendocrine pathways, the autonomous nervous system and the gastrointestinal tract [1]. This network is suspected to influence the development of functional gastrointestinal disorders such as inflammatory bowel disease (IBD), gastroesophageal reflux disease and irritable bowel syndrome (IBS) [1,2,3]. Inflammatory bowel disease, comprising Crohn’s disease and ulcerative colitis, is a chronic, relapsing-remitting inflammatory disorder [4,5]. IBD most commonly presents with symptoms such as abdominal pain, weight loss, diarrhea, anaemia and fatigue [4,5]. Although the disease is prevalent in areas with high socioeconomic status, such as Europe, North America and Oceania, increased incidence rates are being identified in developing countries [6]. Current evidence shows that the prevalence of IBD increased by one third in the last three decades [7]. Genetic, environmental, immunological and microbial factors are involved in disease pathogenesis, indicating a complex etiology [4,8].

Several studies have suggested that psychological factors may influence the IBD course [9,10,11]. Recently, the bi-directional relationship between psychological morbidity and inflammatory activity has received considerable interest [12]. An increasing number of patients with IBD experience comorbid mental health problems, mainly anxiety and depression, affecting considerably their quality of life (QoL) [13,14,15]. Higher rates of depression and anxiety are reported in patients with IBD as compared to healthy controls [16]. A systematic review reports a prevalence rate of 35% for anxiety and depression in these patients [17]. Despite this evidence, psychological comorbidities remain under-recognized and inadequately treated, increasing the psychological burden of the disease [18,19,20]. Additionally, there is a lack of evidence regarding the exact mechanisms by which depression, anxiety and cognitive dysfunction occur in these patients [21]. Published data have recently proposed the microbiota-gut-brain axis, a communication system comprised of bi-directional interactions between the gut microbiota and the brain, as a key element for explaining the association between psychological distress and IBD [22]. Experimental mouse models of colitis have shown behavioral deviations characterized by new-onset depressive and anxiety symptoms after the induction of gut inflammation, caused by the activation of the hypothalamic-pituitary-adrenal (HPA) axis and the immune system [23,24]. Evidence from clinical studies indicates that perceived stress and severity of psychiatric symptoms increase the risk for IBD and relapses, and active disease is associated with both depression and anxiety [25,26].

Psychological disorders represent an important aspect of morbidity and impaired QoL in the IBD population; however, depression and anxiety continue to be under-diagnosed in these patients [27]. In this comprehensive review, we summarize the role of the brain-gut axis in the psychological functioning of patients with IBD, and discuss current preclinical and clinical data on the topic and therapeutic strategies potentially useful for the clinical management of these patients.

### 1.1. Search Strategy

We reviewed and searched MEDLINE and EMBASE using the following terms: “Inflammatory Bowel Diseases”, “Colitis, Ulcerative”, Crohn Disease”, “Depression”, “Anxiety”, “Stress, Physiological”, “Mood Disorders” to identify relevant publications, unrestricted by article type, describing the implication of the brain-gut axis in psychological well-being in patients with IBD in both animal and human studies, the association between mental health disorders and disease course, and therapeutic applications that could potentially be used in the management of the disease. We conducted our search for articles from inception until 21 October 2020, including only articles published in English. Out of 3021 total citations that were identified initially (MEDLINE: 2208; and EMBASE: 813), we selected publications suitable for this review on the basis of importance and emerging concepts in respect to the involvement of the brain-gut axis in the psychological well-being of patients with IBD and translational implications for the disease course.

### 1.2. Psychological Functioning and the Brain-Gut Axis in IBD

Psychological stress induces a local inflammatory response in the gastrointestinal tract, increases intestinal permeability and modifies visceral hypersensitivity and motility [2,3]. Increased intestinal permeability facilitates the bidirectional link between the brain and the gut by means of neural, endocrine, immune, and humoral links [28]. It is now well assumed that the gut microbiota can modulate the blood–brain barrier permeability [29]. The activation of the HPA axis is considered pivotal in mediating the effect of psychological disorders on gut functioning [1]. Stress acts directly on the hypothalamus, stimulating the secretion of the corticotropin-releasing factor (CRF) and subsequently the release of adrenocorticotropic hormone (ACTH) from the anterior pituitary gland [3]. In experimental models of colitis, CRF and ACTH increased the intestinal permeability by inducing mast cell degranulation and cytokine secretion [30,31]. Additionally, stress has a dual, opposite influence on the autonomous nervous system by activating the sympathetic nervous system and by inhibiting the vagally mediated anti-inflammatory effects [3]. During stress responses, the sympathetic nervous system, through the secretion of catecholamines from adrenal medulla, exhibits pro-inflammatory effects in the gastrointestinal tract via activation of mast cells, macrophages through the nuclear factor kB signaling pathway and cytokine secretion [32,33,34]. Stress also disrupts the mucosal barrier, allowing gut microbiota to migrate to secondary lymphoid organs and stimulate an innate immune response and interact with the nervous system [1,35]. Animal studies show an amplified HPA axis response to stress in germ-free mice that is reversed by reformation of the gut microflora, while probiotics lessen behavioral deviations related to alterations of the gut microbiota [36,37,38]. Furthermore, increased intestinal permeability leads to an abnormal enteric nervous system response, i.e., visceral hypersensitivity, with transmission of painful stimuli from gut to brain [1]. A mechanism of visceral hypersensitivity in IBD has not been clearly established yet; however, a possible reason for this is the enteric nervous system activation caused by the exposure to lipopolysaccharides of the local gut bacteria with the subsequent afferent nerve stimulation and activation of the brain–gut axis [1,39]. Comorbid psychologic disorders are also associated with IBS-like symptoms in patients with IBD [40,41].

## 2. Preclinical Studies

### 2.1. Brain-Gut Signaling

Preclinical studies provide the proof of concept that behavioral alterations, such as the induction of depressive and anxiety states, result in hyper-activation of the CRF-mediated hypothalamic pituitary axis and an increased vulnerability to inflammatory gut conditions [42]. A mouse model detected an increased intestinal permeability in maternally separated mice that were in depressive state. After colitis was induced by dextran sodium sulphate, intestinal inflammation was more severe in separated mice than non-separated control mice [43]. Other animal models also showed that inducing depression in mice and rats increased the vulnerability to experimental colitis, indicating a depression-mediated increase in intestinal permeability and attenuation of the vagal anti-inflammatory system [44,45]. Besides these aspects, psychological stress and depression can potentially precipitate a relapse of a quiescent intestinal inflammation [46,47,48]. Intraventricular administration of reserpine, a drug depleting brain monoamine, induces a depressive state in mice with preexisting experimental colitis, resulting in disease reactivation [48,49]. Further preclinical evidence supporting the influence of psychological disorders on the natural history of IBD has focused on the ability of antidepressants to attenuate the inflammatory process [44,48,50,51]. In maternally separated mice with depression-like behavior, treatment with the tricyclic antidepressant desipramine decreased the severity of induced colitis in comparison to non-maternally separated mice with normal behavior [50]. The same result was observed in reserpinized mice with colitis after treatment with desmethylimipramine, which attenuated the duration of immobility associated with depressive behavior [44]. However, the effect was absent in vagotomized mice, suggesting the restoration of parasympathetic function by tricyclic antidepressants (TCAs) and subsequent suppression of inflammation as a possible explanation for this finding [44]. Previous animal studies have also demonstrated a direct anti-inflammatory effect of TCAs [51,52,53,54]. In fact, the administration of fluvoxamine and venlafaxine reduced the colonic inflammation not only in reserpinized rats, but also in non-reserpinized controls [51].

### 2.2. Gut-to-Brain Signaling

Evidence from preclinical studies suggests that intestinal inflammation can influence the behavior and the brain activity. Experimental studies of animal models with colonic inflammation induced by dextrane sulfate sodium detected a behavioral deviation and cognitive impairment in diseased rats [37,55,56,57]. This finding was observed also in vagotomized rats, thus suggesting the mediation by increased circulating TNF-a levels rather than by the activation of afferent vagal pathways [55,58]. A study of chronic colitis that induced an anxiety-like behavior in rats showed increased TNF-a levels and concurrent suppressed expression of brain-derived neurotrophic factor [55]. The administration of anti-TNF-a rescued the anxiety-like symptoms though expression of the brain-derived neurotrophic factor remained low [55]. Moreover, gut inflammation is associated with hippocampal alterations related to inflammation gene expression and glutamatergic transmission, resulting in an increased brain excitability and brain disfunctions [59,60]. Serotonin is suspected to act as a key neurotransmitter in gut-to-brain signaling. The gut microbiota can influence the tryptophan metabolism and the serotonergic system as several bacterial species can metabolize tryptophan as a precursor for the synthesis of serotonin, thus limiting the availability of tryptophan [61].

### 2.3. The Intestinal Microbiota

The human intestine is colonized by a huge number of commensal microorganisms which contribute to maintain the gut integrity, regulate the human immunological system and protect from pathogens: the microbiota [62]. The intestinal microbiota has been recently recognized as an integral part of the brain–gut axis, which can potentially modulate behavioral and cognitive functioning through endocrine, neural and metabolic signaling [55,63,64,65]. Stress and behavioral alterations increase the intestinal permeability, rendering gut bacteria capable of translocating to peripheral lymphoid organs and eliciting an innate immune response [35,66]. An altered microbiota has been long proposed as among the etiological factors of IBD because patients often exhibit a decreased bacteria diversity, particularly in Firmicutes and Bacteroides, and an abundance of Enterobacteriaceae [67]. Gut microbiota alterations of rodents by oral administration of the bacterial pathogen *Campylobacter Jejuni* was found to increase parasympathetic activity and induce an anxiety-like behavior [68]. Short-chain fatty acids, the main product of the dietary fibers by the commensal microflora, are suspected to affect psychological functioning through the G protein-coupled receptors or histone deacetylases and through hormonal and immune pathways and neural networks [69]. Based on this evidence, intestinal microbiota could play a role in promoting intestinal inflammation and may affect the onset and natural history of IBD; however, it is still uncertain whether the alterations observed may be the cause or the consequence of disease onset [67].

## 3. Human Studies

### 3.1. Epidemiology of Psychological Comorbidities in IBD

Depression and anxiety are prevalent among patients with chronic disorders characterized by a strong immune component, such as diabetes, cancer, multiple sclerosis and rheumatoid arthritis [70,71,72,73,74]. Previous studies have demonstrated that depression and anxiety are twice as prevalent in IBD individuals than in the general population [13,75,76,77]. About 15% and 20% of patients with IBD suffer from depression and anxiety, respectively, with some studies reporting prevalence exceeding 30% [17,75,78]. Patients with IBD have lower QoL than healthy individuals, especially those with high disease activity (i.e., not adequately controlled by disease-modifying treatments) and Crohn’s disease [79,80]. Other studies also identified high disease activity as a risk factor for poor psychosocial outcomes in IBD individuals [81,82,83,84].

Despite the rising prevalence of IBD worldwide and its psychological burden, mental health disorders remain under-diagnosed in most patients with IBD [19,20]. In a cohort of 242 patients with IBD who completed the Structured Clinical Interview for Diagnostic and statistical manual of mental disorders, 40% (*n* = 97) and 30% (*n* = 74) of patients met the criteria for a diagnosis of depression and anxiety disorder, respectively [27]. Male sex was the only factor associated with undiagnosed depression or anxiety disorder (OR: 3.09; 95% CI: 1.31–7.30), and patients with undiagnosed anxiety disorder were two times more than those with undiagnosed depression [27]. These findings show a trend of under-recognition and treatment of anxiety disorders, especially due to diagnostic difficulties and reduced efficacy of management options [85,86,87,88].

### 3.2. Antecedent Psychological Comorbidity and IBD Outcomes

Depression can predispose to chronic immune-mediated diseases by activating the immune system through an altered vagal response and increased production of systemic cytokines, thus potentially playing a role in the pathogenesis of IBD [89,90,91,92]. In a large prospective cohort study of 121,700 registered nurses, there was a two-fold increase in the risk of Crohn’s disease, but not ulcerative colitis, in participants suffering depressive symptoms. A more recent diagnosis increased the magnitude of the association [25]. The Manitoba IBD cohort detected an onset of anxiety disorder more than 2 years before the diagnosis of IBD in 64% of individuals, while 54% had experienced a first episode of depression more than two years before the onset of IBD [14]. Another study observed elevated incidence rates of depression and anxiety disorders even 5 years before IBD diagnosis in patients with IBD compared to matched controls [93]. The same conclusion was also drawn from a recent study of 403,665 cases of depression, which demonstrated that depression was associated with Crohn’s disease (HR: 2.11; 95% CI: 1.65–2.70), as well as ulcerative colitis (HR: 2.23; 95% CI: 1.92–2.60), an effect that was mitigated by the use of antidepressants [94].

Although there are many observational studies investigating the association between depression and anxiety and disease course of patients with IBD, it has not been confirmed yet if psychological disorders are responsible for the deterioration of the disease, or that actually the exacerbation of the disease worsens the psychological status of patients [95,96,97]. In a recent systematic review with meta-analysis assessing the association between depressive state and worse disease course, no clear relationship was detected, with conflicting results among the included studies [98]. Most studies including patients with Crohn’s disease demonstrated a stronger influence of depression on the deterioration of the disease as compared with those including patients with ulcerative colitis, thus indicating a possible role of the IBD subtype on the association between depression and disease course [98]. However, patients with active disease and depressive state at baseline were more likely to experience exacerbation of the disease as compared with those in remission [98]. A large longitudinal study of a Swiss IBD cohort reached similar results, reporting a significant association between depression (weaker with anxiety) and earlier clinical recurrence, especially in patients with Crohn’s disease [99]. The association of depression and anxiety with reduced QoL was described also by other long-term prospective studies; however, the generalizability of these findings is limited by the small sample sizes and specific patient characteristics [100,101]. Three prospective observational studies including only patients with ulcerative colitis in clinical and endoscopic remission identified short-term stress [102], long-term stress [82] and an increasing number of stressful events [103] as predictive factors for exacerbation of the disease, thus proposing a psychological basis behind the disease course. Conversely, evidence regarding the role of baseline histologic inflammation in precipitation of relapse remains still controversial [82,102,103]. Further studies underline the intense responses of patients with IBD to psychosocial stressors and the increased risk for disease flare-ups in those with high levels of novel perceived stress and occurrence of life events [34,104]. The main obstacle to establish a temporal relationship between psychological well-being and altered disease course is the lack of objective evaluation of the multiple aspects that disease activity encompasses, and the small sample sizes of the existing observational studies [1].

### 3.3. IBD and Development of Psychologic Disorders

Evidence is accumulating regarding the increased incidence of anxiety, depression and bipolar disorder in patients with IBD [105]. Depressive symptoms were strongly associated with IBD (OR: 3.1; 95% CI: 1.6–5.9) in a large population-based study in the USA [106]. Risk factors independently associated with the increased incidence of depressive symptoms were IBD and a higher number of comorbidities [106]. Generalized anxiety disorder was twice as prevalent in IBD individuals than in healthy controls after adjustment for confounding factors such as sociodemographics, ACEs, depression, substance abuse and pain [107]. Anxiety and depression in patients with IBD, especially during the active phase of the disease, warrants a systematic assessment and management of mental health problems [16,17].

Evidence from observational studies portray an association between active disease and depression, possibly mediated by biases in patients’ neurocognitive processes, although the mechanism still remains unknown [108]. Due to the predominantly early onset of IBD (i.e., second and third decade of life) and the chronic relapsing nature of the disease, severity of disease symptoms influence the psychological well-being and the QoL of patients with IBD [84]. Disease severity may be a determinant for impaired psychological functioning [83,109,110]. Longitudinal studies described an increased incidence of depression and anxiety in patients with worsening disease course over time [75,96,111]. However, an effect of IBD on multiple mental health aspects was observed in another study without an active disease pattern being significantly associated with worse psychological well-being [112]. Furthermore, perceived stigma, a detrimental psychological effect of chronic diseases, is highly prevalent among the IBD population [113]. There were not any significant differences between those with active disease or remission, although patients experiencing frequent flare-ups and more aggressive disease reported higher levels of perceived stress [113].

### 3.4. The Gut-Brain Axis: A Bi-Directional Phenomenon?

It is unclear whether psychiatric co-morbidities directly influence disease activity by exacerbating inflammation, or whether the negative outcomes are indirectly caused by medication, non-adherence to medication or a generally worse subjective feeling that is ascribed to IBD without the presence of objective inflammation [12]. An increased likelihood to develop new onset GI symptoms was noted in patients with high baseline anxiety levels, while patients with functional dyspepsia and IBS, and no mood disorders at baseline, were more likely to develop symptoms of anxiety and depression [114,115]. As mentioned above, animal studies have detected the onset of behavioral changes in experimental models of colitis, while the induction of a depressive state in mouse models with quiescent chronic intestinal inflammation is associated with relapse of the disease that can be reversed by the use of antidepressants [44,48,50,55].

Studies in humans have shown that depression and anxiety can precede the onset of IBD and other immune-mediated diseases for years, which implies a common immune-mediated pathway for psychiatric and somatic inflammatory diseases [93]. Acute psychological stress in patients with ulcerative colitis increased the expression of cytokine and proinflammatory mediators that predispose disease exacerbation, whereas vagal stimulation for 6 months was associated with clinical, endoscopic and biologic remission in patients with active IBD [116,117]. Currently, only two observational studies have examined the bidirectionality of the brain-gut axis in IBD [12,118]. Gracie et al. demonstrated a significant association between an increased baseline clinical disease activity and the development of abnormal anxiety scores (HR: 5.77; 95% CI: 1.89–17.7), while this effect was not visible with depression scores [12]. Similarly, increased baseline anxiety levels were associated with glucocorticoid prescription or exacerbation of the disease (HR: 2.58; 95% CI: 1.31–3.30), confirming the role of brain-gut interactions in patients with IBD [12]. The second observational study reported a bi-directional association between perceived stress and symptom activity [118]. The findings of these studies underline the importance of the brain-gut axis and its influence on IBD course, suggesting potential implications of novel management strategies to reduce the psychological burden of IBD.

### 3.5. Psychological Well-Being and Healthcare Utilization in Patients with IBD

IBD reflects a spectrum of chronic diseases that is associated with increased morbidity, hospital stay and readmissions, resulting in high inpatient costs and care complexity [119]. However, limited data exist evaluating the risk factors for healthcare utilization in patients with IBD and the influence of psychological comorbidities on the disease course [26,120]. Two longitudinal studies identified a significant association between baseline depression and worse disease course over time, including an earlier appearance of more aggressive disease compared with non-depressed patients [121,122]. An internet-based cohort study showed a significant association between baseline depression scores and subsequent disease activity (OR: 1.21; 95% CI: 1.07–1.36), as well as with the number of consequent hospitalizations (OR: 1.26; 95% CI: 1.06–1.49) [123]. However, the lack of validated tools for diagnosis of psychiatric disorders in these patients and objective determination of disease activity cannot confirm the impact of psychiatric comorbidities on the use of healthcare services [48,122].

According to a meta-analysis, pain control was identified among the most significant risk factors for 30-day readmission of patients with IBD [124]. In a cohort study, pre-existing diagnosis of depression or anxiety was associated with increased risk for surgery in patients with Crohn’s disease (OR: 1.28, 95% CI: 1.03–1.57), a finding that was most notable with anxiety disorders [26]. Additional findings of this study included increased healthcare utilization (abdominal CT scans, colonoscopic evaluations) and increased medication use (steroids, immunomodulators, anti-TNF agents) among patients with Crohn’s disease with co-existing psychiatric disorders, while the number of all-cause hospitalizations was increased among patients with ulcerative colitis and Crohn’s disease with such disorders [26]. Retrospective cohort studies have identified the role of psychiatric comorbidities as predictors for unplanned admissions 30 or 90 days after an index admission [120,125]. Among 324 patients with IBD, 102 experienced at least one unexpected readmission 90 days after the initial admission, and depression and chronic pain were risk factors for repeated hospitalizations [120].

A common problem arising in the treatment course of patients with IBD is non-adherence to anti-TNF agents, which is estimated to be from 17% to 45% and is associated with an increase in healthcare utilization and health services cost [126,127,128]. Factors that are associated with increased non-adherence rates in patients with IBD treated with anti-TNF agents include female gender, smoking, treatment beliefs and illness perceptions, along with psychological comorbidities, especially depression and anxiety [126,127,129]. A cohort study including 246 patients with active disease and depression within 30 days prior to the initiation of anti-TNF treatment showed that increased baseline depressive symptoms assessment score was significantly associated with a higher probability of non-compliance with anti-TNF therapy over 2 years of follow-up (HR: 2.28; 95% CI: 1.1–4.6) [130]. An important finding of this study was that depressive symptoms in patients who stopped anti-TNF treatment due to non-compliance were more severe than those who terminated due to medical reasons [130]. In another prospective study of patients with active Crohn’s disease, major depressive disorder at baseline was an independent risk factor for failure to achieve quiescence of the disease after treatment with the anti-TNF agent infliximab, as well as for retreatment compared to patients without major depressive disorder [131]. However, the effect of psychiatric disorders on non-compliance to IBD treatment has been observed not only with anti-TNF agents, but also with aminosalicylates and immunomodulators [132,133,134]. These findings expand the current evidence suggesting the association of psychologic factors with treatment non-compliance in immune-mediated disorders [135].

## 4. Translational Implications with Therapeutic Applications on the Brain-Gut Axis

### 4.1. Pharmacologic Management

Currently, the available treatment options for patients with IBD focus mainly on symptomatic improvement and the induction of remission in patients with active disease, whereas scarce attention is given to psychological wellbeing and QoL [136]. Therapeutic regimens based on 5-aminosalicylates, corticosteroids, biologic agents and immunosuppressive drugs are used during the induction and maintenance phase of the disease to attenuate gut and systemic inflammation [137]. The brain-gut axis and its involvement in psychological comorbidity in patients with IBD outlines the need for new management strategies to improve QoL [12,138].

Depression and anxiety disorders activate an immune response, increasing the production and secretion of various inflammatory markers, including cytokines, adhesion molecules and chemokines [139,140]. An important marker of inflammatory response in depressed patients is TNF-a [141]. Anti-TNF agents have been shown to improve psychological well-being in patients with psoriasis, cancer and Crohn’s disease [131,142,143,144]. A randomized controlled trial (RCT) for treatment-resistant depression did not show any beneficial effect overall, although patients with high C-reactive protein (CRP) at baseline appeared to have greater improvement in depression scores [145]. Patients with baseline CRP > 5 mg/L, treated with infliximab, had significant improvements in various aspects of depressed mood, such as psychomotor retardation, anhedonia, psychic anxiety and suicidal ideation [145].

Psychological disorders promote a proinflammatory state [146]. Treatment with an anti-TNF agent or an immunomodulator for 1–6 months can improve the depressive state of patients with Crohn’s disease regardless of the disease activity [147]. Moreover, poor quality of sleep is a frequent extra-intestinal manifestation in patients with IBD [148], which is mainly bi-directional, with the inflammatory state in IBD disrupting the normal sleep patterns of patients [149,150]. Abnormal sleep increases disease activity and the risk for possible flare-up of the disease [151]. In a prospective cohort study of 183 enrolled patients with IBD, treatment with anti-TNF agents or vedolizumab resulted in improvement of depression, sleep and anxiety within 6 weeks of initiation of treatment up until one year or more [148].

Antidepressants (SSRIs and TCAs) are frequently prescribed to patients with IBD [152]. It is estimated that about one fourth of them use antidepressants and anxiolytics/sedatives respectively [153]. Factors associated with the increased use of psychotropic medications in patients with IBD include Crohn’s disease, middle age, history of gastrointestinal surgery and increasing number of inpatient and outpatient events [153]. Antidepressants exert their anti-inflammatory properties by reducing the production of proinflammatory cytokines (IL-1β, IL-10, IL-4 and TNF-a) and downregulating the expression of nuclear factor kB, which are hypothesized to play a key role in IBD pathogenesis [154]. Another proposed mechanism of the possible beneficial role of antidepressants on the course of IBD is the enhancement of the vagal anti-inflammatory function, which has been observed in an animal model of colitis after treatment with amitriptyline [44].

A systematic review of 15 studies demonstrated a beneficial effect of antidepressants on IBD course, as well as on the decrease of depression and anxiety levels in most included studies, highlighting the possibility of their implication in the current management plans of patients. In an observational study of 67 patients with IBD with increased baseline anxiety, treatment with antidepressants for 6 months resulted in a significant improvement of depression, anxiety, QoL and sexual dysfunction; however, most participants were in remission before the initiation of antidepressant treatment [155]. Frolkis et al. detected that depression and anxiety increased the risk of IBD development, an association that was attenuated by the use of SSRIs and TCAs [94]. Moreover, in a retrospective cohort study including patients with IBD in remission reporting abnormal anxiety/depression baseline levels, use of SSRIs at baseline resulted in lower rates of therapy escalation as compared to those not receiving them (HR: 0.47; 95% CI: 0.24‒0.93) [156]. Similar results were observed for any class of antidepressants (HR: 0.59; 95% CI: 0.35‒1.00); however, the association disappeared after adjusting for confounding [156]. Two RCTs that evaluated the influence of antidepressant use on IBD courses have shown contradictory results [157,158]. In the first one, the authors observed that treatment with duloxetine for 12 months significantly reduced depression and anxiety levels, as well as the mean score of symptom severity as compared to placebo [157]. However, a pilot RCT failed to identify any benefit of treatment with fluoxetine in QoL and symptoms of anxiety and depression of patients with IBD [158].

Gastrointestinal symptoms, such as diarrhea and abdominal pain, are highly prevalent among patients with IBD even without active disease, mimicking the clinical presentation of individuals with IBS and impairing their QoL [159,160]. According to previous meta-analyses, TCAs show beneficial effects in patients with IBS, inducing a significant clinical improvement and decrease in abdominal pain scores [161,162]. The role of TCAs in the management of IBS-related symptoms in patients with IBD was investigated by a retrospective cohort study comprising 81 IBD and 77 IBS patients [163]. A moderate improvement of symptoms was observed in both the IBD and IBS cohorts. A significantly better clinical response of patients with ulcerative colitis was noted as compared to those with Crohn’s disease (83% vs. 50%, respectively; *p* = 0.01) [163]. These findings indicate a promising use of TCAs in patients with IBD with accompanying gastrointestinal symptoms [163].

### 4.2. Psychological Therapies

Besides the use of antidepressants and conventional pharmacologic therapy, a recent clinical practice update recommends psychological interventions, such as cognitive-behavioral therapy, hypnotherapy and mindfulness therapy, for the management of IBD individuals with functional gastrointestinal symptoms [164]. A systematic review with meta-analysis of 32 RCTs has detected a beneficial effect of specific psychological treatments in patients with IBS, mainly cognitive-behavioral therapy, hypnotherapy, dynamic psychotherapy and multi-component psychotherapy either in person or by telephone [165].

Evidence from a previous systematic review regarding the efficacy and methodological challenges of psychotherapy in patients with IBD suggest that cognitive-behavioral therapy can be used as an adjunctive treatment for depression and anxiety in IBD individuals, while hypnotherapy may improve the physical symptoms of the disease and stress coping strategies need more evidence [166]. However, an RCT with weekly 2 h cognitive-behavioral therapy sessions delivered either face-to-face or online did not have a significant effect on disease activity of IBD participants after 24 months of follow-up, and did not improve their mental health state [167]. A similar finding was seen in another RCT evaluating the impact of multi-convergent therapy (cognitive-behavioral therapy techniques with mindfulness-meditation) on the IBDQ scores, in which the improvement was not statistically significant in the intention-to-treat population; however, after a subgroup analysis involving patients with IBD with IBS-related symptoms, a statistically significant improvement in QoL was observed [168].

Another psychological intervention that has been evaluated and implicated mainly in the treatment of IBS, is gut-directed hypnotherapy [169]. The mechanisms by which hypnotherapy exerts its beneficial effects in gut diseases include anti-inflammatory properties, alterations in central processing of peripheral visceral signs and effects on the autonomous nervous system [169]. A systematic review of seven RCTs reported a significant improvement in gastrointestinal symptoms of IBS patients in six studies; the effect remained long-term in four studies. In this review, only one RCT discussed the role of gut-directed hypnotherapy in IBD individuals [169]. Patients with ulcerative colitis remained in remission for significantly more time as compared to controls, and this difference was still significant after one year [169,170].

Stress coping strategies, and especially mindfulness-based stress reduction, are used as supplemental treatments for anxiety disorders and have been shown to induce physical and psychological benefits in chronically ill patients and patients suffering from chronic pain and fibromyalgia [171,172,173]. A trial including patients with ulcerative colitis in remission examined the influence of mindfulness-based stress reduction therapy on disease course, psychological well-being and QoL [174]. No effect of the psychological intervention was observed on disease course and inflammatory markers, whereas it improved QoL in patients who experienced a relapse [174]. Two other RCTs examining the effect of mindfulness-based stress reduction therapy have detected an improvement in QoL and depression scores; however, no change was observed on disease course, disease activity, and various inflammatory markers of the disease [175,176].

The most recent study that evaluated the influence of psychological therapies on disease course, QoL, mental health and perceived stress of patients with IBD is a systematic review and meta-analysis by Gracie et al., including 14 RCTs and 1196 patients. Most patients were in remission (only two RCTs included patients with active disease) and received cognitive-behavioral therapy, psychodynamic psychotherapy, stress-reduction treatments or hypnotherapy. The results showed that psychological therapy did not have beneficial effects on disease course and mental health scores (anxiety, depression, perceived stress) of patients with IBD. However, psychological interventions induced a significant improvement in disease-related QoL at the end of therapy that was lost at the end of the follow-up. This effect was more prominent with cognitive-behavioral therapy treatment, while no significant benefit was observed in study outcomes according to IBD subtype. To determine the effect of psychological interventions on IBD course, as well as psychological functioning of patients with IBD, further, adequately powered RCTs should be conducted, which should take into account baseline disease activity status and consider the frequent drop-outs that occur in psychological treatments [166].

### 4.3. Potential Therapies Targeting the Microbiome

Among the different therapeutics that can potentially be used to target the microbiome, probiotics is the most commonly studied in the literature. Probiotics exert anti-inflammatory properties in murine models of colitis and maintain the integrity of the gut barrier, rendering them as potential agents in the treatment of IBD [177]. According to experimental studies, consumption of probiotics can be helpful in the management of depression by downregulating the HPA-axis that is highly activated in depressed patients and by increasing the production of GABA and serotonin, neurotransmitters with antidepressant properties [178,179,180]. Although more evidence exists regarding the management of IBS (psychological interventions, diet, probiotics) [181], a recent meta-analysis of 22 RCTs evaluated the role of probiotics in the management of IBD individuals [182]. The results showed no additional benefit of probiotics as compared to placebo in inducing remission in patients with active ulcerative colitis and equivalent action to ASAs in preventing relapse of the disease [182]. When the studies examining the probiotic VSL#3 were analyzed separately, there was a significant benefit for patients with active ulcerative colitis (RR: 0.74; 95% CI: 0.63‒0.87) [182]. However, in patients with Crohn’s disease, probiotics did not exert a beneficial effect in preventing relapse of the disease, even after surgically inducible remission, or bringing the disease to a quiescent state [182].

Flatulence, bloating, diarrhea, constipation and abdominal pain are common symptoms in patients with IBS, functional GI disorders and IBD that impair their QoL [183]. Even patients with IBD in remission experience gastro-intestinal symptoms that fulfill the criteria for concurrent diagnosis of IBS [183,184]. New evidence suggests that a diet high in FODMAPs (Fermentable, Oligosaccharides, Disaccharides, Monosaccharides and Polyols) is responsible for generating abdominal symptoms in patients with IBS-like symptoms and IBD [185,186]. FODMAPs are poorly absorbed short-chain carbohydrates that stay in the gut lumen and are fermented by colonic bacteria in gas products that trigger the abdominal IBS-like symptoms [187,188]. Clinical evidence suggests that a low FODMAP diet exerts a beneficial effect in the symptoms of patients with IBS and currently is indicated in the management of the disease [189,190,191]. In a RCT of 78 patients with IBD with IBS-like symptoms in remission or with mild-to-moderate disease, a low FODMAP diet for 6 weeks resulted in significant reduction of IBS symptoms and improvement of QoL as compared to patients that followed a normal diet [192]. Following a subgroup analysis, the results showed greater benefit in symptoms improvement in patients with Crohn’s disease with a history of bowel surgery and in those with quiescent disease, while a trend toward reduction of disease activity was seen in patients with ulcerative colitis [192]. An RCT of 88 patients investigating the efficacy of low FODMAP diet in IBD individuals with functional gastro-intestinal symptoms noted a significant control of symptoms in the majority of patients and a reduction in reported symptoms of any severity, such as abdominal pain, flatulence, bloating, incomplete evacuation or heartburn, as well as improvement in stool consistency in most patients [193]. In a randomized, double-blinded, placebo-controlled, cross-over, re-challenge trial, 32 patients with IBD followed a low FODMAP diet with adequate relief of their symptoms [194]. Patients were randomly assigned to 3-day FODMAP challenges with subsequent assessment of stool output and symptom severity [194]. There was a significant increase in incidence and severity of IBS-related symptoms in the fructan challenge group as compared to the placebo group (glucose), findings that were not observable in sorbitol and galacto-oligosaccharides challenge groups [194]. Two other studies have shown an improvement on symptoms of abdominal pain, bloating and diarrhea in IBD individuals, but not on constipation in which the response was inadequate [195,196]. Even though there are clinical studies, and especially RCTs showing a beneficial effect of low FODMAP diet in IBS-related symptoms in patients with IBD, larger studies should be conducted in order to introduce this novel strategy in patient management.

Given the role of fecal microbiota in the pathogenesis of IBD, another management strategy that has gained ground recently is fecal microbiota transplantation [197]. Fecal microbiota transplantation has demonstrated high efficacy in the treatment of recurrent Clostridium difficile infection with inadequate response to standard treatment [198]. In a meta-analysis of patients with active ulcerative colitis, a higher proportion of patients receiving fecal microbiota transplantation achieved combined clinical/endoscopic remission as compared to those receiving placebo with a good safety profile [199]. However, the effect was short-term and further studies are needed to prove the efficacy of fecal microbiota transplantation as a maintenance treatment and establish the safety of the procedure in order to be introduced in the treatment of patients with IBD [199].

### 4.4. Environmental Factors Affecting the Brain-Gut Axis

The epidemiology of IBD has been evolving over the last few years with an increasing adoption of the Western lifestyle [200]. Other than for predisposing genetic factors, dysbiosis, diet changes and environmental risk factors from the early life period play a pivotal role on the onset of this spectrum of diseases [8,200]. Especially, the phenomenon of “urbanization”, which includes behavioral changes, diet alterations and exposure to environmental pollution, which might affect the development of IBD in the Western world, and evidence has shown an increased incidence of Crohn’s disease and ulcerative colitis in urban societies [201,202]. In the last few decades, urban environments are characterized by high levels of ambient air pollution which has serious health effects in residents of these areas. From the perspective of the effects in the gut microenvironment, air pollution activates the innate immune system and increases the secretion of pro-inflammatory cytokines, while concurrently disrupting the gut barrier, creating an inflammatory state that alters gut microbiota [203]. Additionally, high concentrations of NO_2_ and SO_2_ in ambient air has been associated with earlier development of Crohn’s disease and ulcerative colitis respectively [204]. An ecological analysis has demonstrated a direct correlation between air pollutant emissions and IBD hospitalizations in the state of Wisconsin, an effect that was also observable in other immune-mediated diseases, such as multiple sclerosis and asthma [205]. This evidence suggests the hypothesis that environmental conditions and gut inflammation are associated with each other, creating new pathways and environmental interventions in prevention strategies of IBD [200,202].

## 5. Putting Research into Context

Depression and anxiety represent the most common psychological comorbidities in IBD individuals and induce detrimental effects in their QoL [21]. It is estimated that over 20% of patients with IBD have depressive symptoms, while anxiety symptoms are prevalent in more than 35% of them, with higher percentages in those with an active disease [17]. Given the increasing diagnosis of mental health disorders in IBD, more animal and human studies are being conducted to evaluate the emerging role of the brain-gut axis, and recently microbiota, in the psychological well-being of these patients [21]. Patients with IBD report high levels of perceived stress and major psychological stressors during the course of their illness that are not addressed optimally by specialists [136]. Specialists give much more importance to reducing inflammation and disease symptoms; however, there is an increasing need to consider multi-faceted interventions for these patients, including their psychological needs [136]. Besides the standard treatments of patients with IBD, learned societies suggest the implementation of integrated healthcare models that should assess the patients holistically with the frequent use of screening tools for the identification of mental health disorders [206].

The microbiota-brain-gut axis has been studied and identified in animal studies of germ-free mice, in which the vagus nerve carry bi-directional neural signals between the gut and the brain and exert protective effects in murine models of colitis [44]. Furthermore, intestinal microflora mediates the production of various neurotransmitters and metabolic products that alter the neural function in experimental conditions [55]. However, implementing the results of preclinical studies in humans is challenging, and no causal association has been clearly established between the brain-gut axis and psychological comorbidities in patients with IBD, as the etiology of anxiety and depression can be attributed to multiple factors [21]. The reason for this is that mainly observational studies have presented an increased prevalence of psychological disorders in patients with IBD and their role on the course of disease, rendering imperative the conduction of RCTs in order to minimize potential bias and limitations and enhance the power of results. RCTs of novel treatments targeting specific parts of the microbiota-brain-gut axis (e.g., antidepressants, psychological therapies, dietary interventions, fecal microbiota transplantation) have shown promising results in the treatment of psychological disorders and IBS-related symptoms in patients with IBD. However, RCTs with larger sample sizes and more robust methodology should be conducted.

## 6. Conclusions

Despite evidence gaps in the role of the brain-gut axis on mental health of patients with IBD, we suggest that an integrated model of care should be promoted as the standard of care in the IBD population, with a patient-centered approach and repeated behavioral evaluations [207]. This is a fundamental step in order to promote precocious diagnosis of alterations in the psychological functioning of fragile patients and taking appropriate steps toward effective and timely therapeutics strategies. Personalized pathways for patients with IBD are needed, as these patients prefer receiving psychological support from professionals understanding their pathological condition without stigma to better elaborate their coping strategies [208]. This approach, accompanied by the upcoming results regarding the efficacy of novel strategies targeting the brain-gut axis, can contribute to a better QoL and psychological well-being of patients. 

## Data Availability

Data sharing not applicable.
